# Mr. Besant's Latest Morality

**Published:** 1889-12-28

**Authors:** 


### Mr. Besant's Latest Morality.

Of all our living novelists none, perhaps, has done more to deserve the esteem—to say the least of it—of the social philosopher than Mr. Walter Besant, not only because his charming novels have so often lightened the weariness which constant wrestling with social problems sometimes induces, and substituted for it a restorative calm "felt in the blood and felt along the heart," but because the general tendency of his works is in the direction in which the social philosopher would wish to see more people moving. As a mere literary workman, Mr. Besant is, undoubtedly, *un homme charmant* in the full force of that term. He has assimilated the best qualities of the French humourists about whom he has written with so much sympathy, and as a painter of contemporary manners he exemplifies what is called a thoroughly good fellow. His books (especially this little one) bewray him, suggesting associations which strengthen with further reading; and their presence becomes as "pleasant as a sudden rush of warm air in winter, or the flash of firelight in the chill dusk." It is very evident that their production affords him decided pleasure; and his dictum that "no young man is so universally beloved as he who can play and sing with ease and freedom, and as if he enjoyed it as much as his hearers," applies, *ceteris paribus*, to himself. In fact, as one reads the description of Kit Cotterel at the piano, one insensibly thinks of the author who created so thoroughly human and lovable a character: "Why, the man was bubbling over with music of the soft and sentimental kind, of the bacchanalian kind, or of the love kind—willingly at all times would Kit sit down to sing hymns ancient and modern in praise of Venus or of Bacchus—of love and wine. And as he sang, his light voice rolling above the rippling of the notes, his face would shine and beam, and his lips would so laugh, and he would be so happy in the exercise of his power that everybody loved him."

The power which can thus cause joy is an invaluable factor for good "amid the fretful stir unprofitable and the fever of the world"; and its value is enhanced when, as in Mr. Besant's case, it is informed by a practical acquaintance with the conditions which make life, especially in London, so miserable for so many of our fellow-creatures, and by a hearty sympathy with the great movements which earnest and thoughtful men are setting afoot for the alleviation and improvement of this life. And it is for this reason that we welcome Mr. Besant's little work,* which it has pleased Mr. Arrowsmith to entitle a "Christmas Annual, 1889." Not that Mr. Besant throws any new and unexpected light on the social problems of the day: his opinions on such subjects are as widely known as his works, and the antithesis (to call it by no stronger name) between himself and Political Economy is no less familiar to all of us. He suggests, indeed, an answer to the question started the other day by the *Standard*, when commenting on Sir Edward Guinness's generous munificence, why it is that rich men who owe their fortunes to their own personal exertions have supplied up to the present almost all the instances of munificence in this direction, while those who have inherited properties from a long line of ancestors, and who, with their properties might be supposed to have inherited a keen sense of the duties which property entails, have, for some reason, been wanting; but the answer to this is equally suggested by our own daily experience. Wordsworth has told us that Man is dear to Man; but we have heard with our ears, and our experience has declared unto us, that this sympathy is only most truly felt by the poorest poor, who

> Long for some moments in a weary life
> When they can know and feel that they have been
> Themselves, the fathers and the dealers out
> Of some small blessings; and that it blossoms into benevolent action among those sturdy, sterling men (of whom we are proud to know that there are many among what are called generically City Men) who, having emerged victoriously from the storm and stress of the battle of life, do their utmost to alleviate the lot of those who are weaker or less fortunate than themselves. Here and there, it is true, a man who has been born in the purple which marks the caste either of Vere de Vere or of Sir Gorgius Midas, has thrown himself with remarkable zest into great popular movements of reform. But these men are as rare as black swans, and it would seem as if the rich man who, like Count Almaviva, had only taken the trouble to be born, must, before he can thus become sympathetically useful to his kind, learn, by his own personal experience, what they actually feel and want. And as Dante found that the way to heaven lay through the dark valley of hell, so too Dives, who wishes to attain the heaven which is tenanted by the great benefactors of the world:

> Necessita il c'induce, e non diletto.

Mr. Besant's little book is, apparently, written to illustrate this thesis; but, as becomes a moralist who only castigates by laughter, he conveys his opinions by means of an allegory which is as delightful in its way as that most charming of all French parables: Alfred de Musset's *Histoire d'un Merle Blanc.*

With regard to this allegory, it is, perhaps, more becoming that we should follow the example of Mr. Andrew Lang when writing to Herodotus "concerning a certain sacred story which they tell in the City of the Ford of the Ox." Suffice it is to say, therefore, that it is a Christmas Morality of the good, old-fashioned type, tempered by the large and kindly tolerance of our own days, in which the principles most congenial to Mr. Besant are personified and contrasted, to their exceeding great advantage, with the representative of a hard, priggish, pedantic Political Economy; and that the thesis is worked out with an accompanying abundance of humour, an accumulating animation, and an unflagging *brio*, that remind us of Edmond About at his best: in *L'Homme à l'oreille cassée*, for instance, or *Le nez d'un notaire*. It is a thoroughly healthy and happy little production: eminently Besantine. And its moral is like unto the author thereof.

* *The Doubts of Dives.* By Walter Besant. Arrowsmith's Christmas Annual, 1889.

---

Much attention has lately been paid in Berlin to the desirability of establishing special hospitals for phthisical patients, chiefly on the ground of the contagiousness of phthisis. As it is a disease the treatment of which requires much skill, special hospitals would doubtless prove valuable.

